# Bioactive glasses and electrospun composites that release cobalt to stimulate the HIF pathway for wound healing applications

**DOI:** 10.1186/s40824-020-00202-6

**Published:** 2021-01-15

**Authors:** Anu K. Solanki, Ferdinand V. Lali, Hélène Autefage, Shweta Agarwal, Amy Nommeots-Nomm, Anthony D. Metcalfe, Molly M. Stevens, Julian R. Jones

**Affiliations:** 1 Department of Materials, Imperial College London, South Kensington, London, SW7 2AZ UK; 2 Institute of Biomedical Engineering, Imperial College London, South Kensington, London, SW7 2AZ UK; 3 Department of Bioengineering, Imperial College London, London, SW7 2AZ UK; 4 The Griffin Institute, Northwick Park & St Mark’s Hospitals Campus, Watford Road, Harrow, HA1 3UJ UK; 5 Healthcare Technologies Institute, School of Chemical Engineering, University of Birmingham, Edgbaston, Birmingham, B15 2TT UK

**Keywords:** Bioactive glass, Cobalt, HIF pathway, Wound healing, Bioactive composites

## Abstract

**Background:**

Bioactive glasses are traditionally associated with bonding to bone through a hydroxycarbonate apatite (HCA) surface layer but the release of active ions is more important for bone regeneration. They are now being used to deliver ions for soft tissue applications, particularly wound healing. Cobalt is known to simulate hypoxia and provoke angiogenesis. The aim here was to develop new bioactive glass compositions designed to be scaffold materials to locally deliver pro-angiogenic cobalt ions, at a controlled rate, without forming an HCA layer, for wound healing applications.

**Methods:**

New melt-derived bioactive glass compositions were designed that had the same network connectivity (mean number of bridging covalent bonds between silica tetrahedra), and therefore similar biodegradation rate, as the original 45S5 Bioglass. The amount of magnesium and cobalt in the glass was varied, with the aim of reducing or removing calcium and phosphate from the compositions. Electrospun poly(ε-caprolactone)/bioactive glass composites were also produced. Glasses were tested for ion release in dissolution studies and their influence on Hypoxia-Inducible Factor 1-alpha (HIF-1α) and expression of Vascular Endothelial Growth Factor (VEGF) from fibroblast cells was investigated.

**Results:**

Dissolution tests showed the silica rich layer differed depending on the amount of MgO in the glass, which influenced the delivery of cobalt. The electrospun composites delivered a more sustained ion release relative to glass particles alone. Exposing fibroblasts to conditioned media from these composites did not cause a detrimental effect on metabolic activity but glasses containing cobalt did stabilise HIF-1α and provoked a significantly higher expression of VEGF (not seen in Co-free controls).

**Conclusions:**

The composite fibres containing new bioactive glass compositions delivered cobalt ions at a sustained rate, which could be mediated by the magnesium content of the glass. The dissolution products stabilised HIF-1α and provoked a significantly higher expression of VEGF, suggesting the composites activated the HIF pathway to stimulate angiogenesis.

**Supplementary Information:**

The online version contains supplementary material available at 10.1186/s40824-020-00202-6.

## Background

Bioactive glasses are those that can provoke a specific beneficial biological response when implanted [1–3]. To date, bioactive glasses have been primarily applied in bone regeneration [4–6] as they have shown osteogenic properties in vitro [7] and the ability to form a strong bond to bone in vivo [5, 8, 9]. A glass was traditionally defined as bioactive if it forms an apatite-like layer on its surface after incubation in Simulated Body Fluid (SBF) [10], since in vivo bonding to bone often occurs following the formation of a hydroxycarbonate apatite (HCA) layer [11]. The use of SBF to predict in vivo results is disputed, due to the conditions used [12] and bioactivity in vivo is more complex, involving many cells and factors. However, testing in fluids can provide useful information on the degradation and ion release properties of bioactive glasses and controlling ion release is key for the therapeutic properties of the glass [13].

An emerging field of regenerative medicine is the use of bioactive glasses in wound healing applications [14, 15], where it has been found that the bioactive glasses can decrease healing times [16–18]. A borate based bioactive glass formed into cotton-like fibres, named MIRRAGEN (ETS Woundcare, Rolla, MO), recently attained FDA approval for indications such as chronic wounds including diabetic ulcers. Early studies on the angiogenic properties of the original 45S5 Bioglass, and its composites, found certain concentrations could promote Vascular Endothelial Growth Factor (VEGF) expression from fibroblasts [19, 20]. Although bioactive glasses are increasingly being studied for wound healing applications, the glass compositions being investigated have previously been shown to form a hydroxycarbonate apatite (HCA) layer on their surface [21]. For wound healing applications, a new glass composition may be required as a HCA layer is not required to facilitate bonding to bone. In fact, HCA formation may inhibit haemostasis [22] and calcium deposits have been shown to impede healing of leg ulcers [23–25].

The use of hypoxia mimicking agents in wound healing has been shown to improve healing times in vivo [26–28]. An important part of wound healing is angiogenesis and cobalt is a known hypoxia mimicking agent, since it activates the HIF pathway by stabilising HIF-1α at ambient oxygen levels. In normal oxygen environments, HIF-1α is constitutively expressed and continually degraded by proteasomes, following hydroxylation by prolyl hydroxylase domain (PHD) enzymes [29, 30]. In hypoxic conditions or in the presence of a hypoxia mimicking agent such as cobalt, the stabilisation of HIF-1α allows the formation of the HIF complex, which is translocated to the nucleus, and leads to an increase in the expression of genes involved in the adaptation to hypoxia. This includes genes involved in angiogenesis such as Vascular Endothelial Growth Factor (*VEGF*) [31]. Cobalt ions released from bioactive glass have been shown to stabilise HIF-1α in human mesenchymal stem cells (hMSCs) [32] and cobalt ions, delivered in the form of CoCl_2_, have increased the production of VEGF by hMSCs [32], endothelial cells [33, 34] and fibroblasts [35].

The ability of a biomaterial to promote the formation of blood vessels could be of major benefit when aiming to heal a chronic wound. A number of approaches have been used to promote angiogenesis, including the incorporation of growth factors, e.g. direct delivery of VEGF, and delivery of other therapeutic ions [36–38]. Benefits of delivering pro-angiogenic ions, such as cobalt, from a bioactive glass are that the cobalt is released as the glass degrades, giving the ability to control the release rate through the glass composition [32, 39–41], and the cobalt ions are released without associated anions. Cobalt has been successfully incorporated into melt-derived silicate glasses, where it has been shown to take a network intermediate role in the glass structure, with the ability to act as both a network modifier and a network former [32, 41–43]. This led to phosphate [44] and borosilicate-based [45] compositions being developed. More recently, cobalt was also incorporated into sol-gel glass [46, 47], provoking VEGF expression from fibroblasts [48], and into inorganic/ organic hybrid scaffolds [49], although its role in the glass network was not confirmed.

Bioactive glass products have traditionally been in the form of a glass particulate [4]. One drawback of particles is the inherent difficulty of containing them in a fixed space which could make them unsuitable for wound healing applications. 3D porous scaffolds [50, 40, 51–54], fibres [17, 18, 21, 36, 37, 55, 56], and melt or electrospun composites [57–59], which are easier to handle than particles, have all been produced with bioactive glasses. Electrospinning has also been used to produce sol-gel [60] and hybrid fibres [61]. Poly(ε-caprolactone) (PCL) [62, 63] has been commonly used to electrospin fibres, with the fibre properties controllable by varying the electrospinning conditions. Inorganic particles such as hydroxyapatite [64, 65], silica doped calcium carbonate [66], calcium phosphates [67], and bioactive glasses [68, 69] have all been incorporated to improve the mechanical and biological properties, with the particle size typically on the nanometer scale. Recently, Co-containing sol-gel glass nanoparticles were incorporated into electrospun PCL, but they nucleated apatite in SBF due to high calcium content [70].

Electrospun fibres have been widely investigated for wound healing applications due to both their high surface area and their ability to both absorb exudate from the wound bed and conform to irregularly shaped wounds [71]. For wound dressings, research has focused on the development of fibres loaded with antibacterial agents such as silver [72] and growth factors [37, 73, 74] to reduce healing times.

The aim of this study was to design new melt-derived, cobalt bioactive glass compositions that do not form a HCA layer on their surface, and degrade at a rate that allows cobalt ions to be released, at a sustained rate, suitable for a therapeutic effect. The strategy for glass design was to incorporate cobalt  (Co) and reduce apatite forming ability, while maintaining the silicate network connectivity of the original 45S5 Bioglass to enable sustained dissolution. Particles of the new glass compositions were incorporated into electrospun PCL fibres, to make the device easier to handle [75, 76]. In vitro assays were conducted with primary fibroblasts to investigate the ability of these materials to stabilise HIF-1α and promote VEGF expression.

## Materials and methods

### Designing of bioactive glass compositions and preparation

Glass compositions were specifically designed for wound healing applications to release cobalt ions as the glass degraded without the precipitation of a HCA layer on their surface. Therefore, the compositions were phosphate-free, and calcium was minimised. Mg^2+^ was used to substitute for Ca^2+^ to reduce calcium phosphate precipitation [77]. Two series of glass compositions were designed to compare the difference between 5 and 2 mol% of cobalt (Table 1). The 2Co glasses had fixed SiO_2_ content and fixed network connectivity. Network connectivity (NC) of a glass is the mean number of bridging oxygen (Si-O-Si) bonds per silicon atom [78]. NC'' was calculated assuming that all intermediates act solely as modifiers (eq. 1), charged balanced by divalent cations [52]:

1 $$ N{C}^{\prime }=\frac{4\left({M}_f\right)-2\left({M}_2^IO+{M}^{II}O\right)}{M_f} $$

where M_f_ is the molar fraction of the network forming oxide, e.g. Si, and ${M}_2^IO$ and *M*^*II*^*O* are the molar fractions of the mono and divalent modifier oxides respectively. The aim was to keep the NC' close to that of 45S5 Bioglass (2.12), as it is known to undergo dissolution in vivo*,* but here the calcium content was reduced compared to 45S5 to slow HCA formation. As magnesium and cobalt are both known to act as network intermediates, NC'' was calculated assuming that 10% of both the magnesium and cobalt act in a network forming role, with 10% an estimation from previous solid state NMR [41, 77], simulations [79] and diffraction studies [42]:

2 $$ N{C}^{\prime \prime }=\frac{4\left({M}_{Si}\right)-2\Big({M}_2^IO+{M}^{II}O+\left(0.9{M}^{Co}\right)+\left(0.9{M}^{Mg}\right)-\left(0.1{M}^{Co}\Big)-\left(0.1{M}^{Mg}\right)\right)}{\Big({M}_{Si}+\left(0.1{M}^{Co}\Big)+\left(0.1{M}^{Mg}\right)\right)} $$

where *M*_*Si*_ is the molar fraction of network forming Si, *M*^*Co*^ is the molar fraction of Co and *M*^*Mg*^ is the molar fraction of Mg.

**Table 1:** Glass compositions used in this study (mol%). Network Connectivity (NC') was calculated assuming that all intermediates acted solely as modifiers, and NC'' assumed that 10% of the intermediates took a network forming role

Glass No.	SiO_**2**_	Na_**2**_O	CaO	K_**2**_O	CoO	MgO	NC'	NC''
**5Co-A**	55	20		10	5	10	2.36	2.41
**5Co-B**	60	20		10	5	5	2.67	2.69
**2Co-A**	50	24			2	24	2.00	2.10
**2Co-B**	50	24	24		2		2.00	2.01

An objective was to develop biodegradable glasses that do not mineralise, and to investigate the effect of substitution of the Ca in the glass for Mg on the rate of Co release and mineralisation. So, composition 2Co-A contained 24 mol% MgO in place of the 24 mol% CaO in 2Co-B. In the 5Co glasses, MgO was partially substituted for silica as MgO is known to be a network intermediate [77], to investigate its influence on the effect on Co release. Higher NC' values were also tested (5Co-A and 5Co-B) in order to slow ion release, wherein magnesium was partially substituted for the silica. K^+^ was also used in the place of some Mg, to reduce the MgO content from 24 mol%.

Precursor materials of SiO_2_ (Prince Minerals), CoCO_3_ (Alfa Aesar), Na_2_CO_3_, MgO, CaCO_3_, and K_2_CO_3_ (all from Sigma Aldrich) were mixed and melted in a Pt-5%Au crucible for 1.5 h at 1450 °C. The molten glass was quenched into deionised water and dried overnight at 120 °C. The glass frit was then ground in a planetary ball mill (Fritsch Pulverisette 7) for 6 min at 500 rpm to produce glass particles with a D_(0.9)_ of between 60 and 80 μm.

### Evaluation of glass dissolution and hydroxycarbonate apatite layer on glass surface

To evaluate the ion release and formation of a HCA layer on the glass surface, glass particles were incubated in Simulated Body Fluid (SBF), which was prepared according to Kokubo et al. [10]. According to an internationally agreed protocol, 150 mg of glass particles were added to 100 ml of SBF and incubated in an orbital shaker at 37 °C, agitating at 120 rpm [80]. At every time point (1, 2, 4, 8, 24 h, 4, 7, 14, and 21 d), pH was measured, and 1 ml of media was collected and replaced with 1 ml of fresh SBF. The 1 ml samples were diluted 1:10 with 2 M HNO_3_ before the elemental concentrations were quantified using Inductively Coupled Plasma Optical Emission Spectroscopy (ICP-OES) (Thermo Scientific, iCAP6000 Series ICP). After incubation, the glass powders were collected in filter paper, rinsed briefly with deionised water and acetone to stop any further reaction, and left to dry overnight.

Fourier Transform Infrared spectroscopy (FTIR) and X-Ray Diffraction (XRD) were carried out on the glass particles before and after incubation in SBF to assess changes in the glass structure and the formation of a HCA layer. FTIR was carried out using a Nicolet iS10 FTIR in Attenuated Total Reflectance mode with a resolution of 0.4 cm^− 1^ in the range of 400–2000 cm^− 1^. XRD was carried out using a PANalytical instrument from 5 to 80 °2θ with a step size of 0.0334225. The particles were observed before and after 21 d incubation in SBF, using a Scanning Electron Microscope (SEM) (LEO Gemini 1525 FEGSEM). Samples were imaged after coating with gold, using an accelerating voltage of 5 kV.

For Transmission Electron Microscopy – Energy Dispersive Spectroscopy (TEM-EDS) analysis, samples were prepared using a focussed ion beam (FIB, FEI Helios NanoLab 600) operated at 30 kV. Briefly, a 15 μm × 2 μm site on the sample was coated with 1.5 μm thick platinum using a current of 93 pA. Two trenches of dimensions 18 μm × 4 μm × 4 μm (length x width x depth) were made on either side of the platinum protected layer using a 2.8 nA current. The base of protected region was then cut using a 6.4 nA current. The section was attached to the omniprobe manipulator by a 0.5 μm thick layer of platinum, lifted out from the bulk sample, and attached to a TEM lift-out 3 post copper grid (Agar Scientific) with a 1 μm thick layer of platinum before being further thinned down to approximately 100 nm in width using currents between 0.46 nA to 2.8 nA. Finally, to remove the possible artefacts introduced by milling, the sample surface was polished with a gallium ion beam operated at 2 kV. Samples prepared by FIB were imaged in the TEM (JEOL JEM 2100F) operating at 200 kV using dark field scanning transmission electron microscopy (STEM) mode. Elemental maps were obtained by EDS (Oxford Instruments INCA EDS 80 mm X-Max detector system with light-element (Z > 5) analysis and STEM capability).

### Electrospinning composites

Preliminary experiments were conducted to determine the optimal PCL concentration in chloroform, and wt% glass particles according to the final scaffold. PCL with a number average molecular weight of 70–90 kDa (Sigma Aldrich) was dissolved in chloroform (Fisher Scientific) at a concentration of 12 wt/v%. Glass particles were added at 30 wt% according to the final weight of the scaffold. The polymer/glass solution was mixed for 1 h, before electrospinning through a 40 mm long 18G needle, onto a grounded mandrel rotating at 100–150 rpm. The tip to collector distance was 10 cm, flow rate was 2 ml h^− 1^, and a voltage of 16 kV was used. The fibres were observed under an SEM (LEO Gemini 1525 FEGSEM) after coating with chromium, using an accelerating voltage of 5 kV.

### Evaluation of ion release from composites

The ion release from the composites was measured in Dulbecco’s Modified Eagle Medium (DMEM). To keep the ratio of glass to media consistent with the SBF study, 50 mg of composite and 15 mg of glass particles were added to 10 ml of DMEM. Samples were incubated in an orbital shaker at 37 °C, agitating at 120 rpm. 0.5 ml of media was collected at each time point (0.5, 1, 2, 4, 8, 24 h, 3 and 7 d) and replaced with 0.5 ml of fresh media. The 0.5 ml sample was diluted 1:20 with deionised water prior to measuring the ionic concentration by ICP-OES.

### Cell culture

Skin was obtained with patient consent and full approval from the National Regional Ethics Service (REC: 06/Q1907/81) from discarded tissue during routine surgical procedures. Primary human fibroblasts were isolated from this and cultured as previously described [81]. Fibroblasts were expanded and maintained in DMEM supplemented with 10 v/v% Foetal Calf Serum (FCS) and Penicillin and Streptomycin at 50 Units ml^− 1^ and 50 μg ml^− 1^, respectively (growth medium). Conditioned medium was prepared by incubating the electrospun composite in low serum medium (DMEM + 1 v/v% FCS + Penicillin and Streptomycin at 50 Units ml^− 1^ and 50 μg ml^− 1^) at a ratio of 1 mg/ml for 24 h, after which the composite was removed and the medium filtered through a 0.2 μm syringe filter. Conditioned medium was prepared immediately prior to use. The growth medium was also used as control media.

### Alamar blue assay

The metabolic activity of fibroblasts was measured using an alamarBlue® kit (Invitrogen, UK). Fibroblasts were seeded at a density of 31,250 cells cm^− 2^ in a 96 well plate, and allowed to adhere overnight before treatment with the conditioned medium for 1, 3 and 7 d. At each time point the alamarBlue® solution was made up according to the manufacturers protocol, added to each well, and incubated for 3 h before measuring the absorbance at 570 nm with a reference wavelength of 600 nm.

### Western blot

Fibroblasts were seeded at a density of 26,315 cells cm^− 2^ in a 12 well plate. Following overnight attachment, the cells were rested in DMEM + 1 v/v% FCS for 72 h and then incubated in 0.5 ml conditioned or control medium for 6 h. The cell monolayer was lysed in 100 μl of laemmli buffer and heated to 95 °C for 5 min before resolving 30 μl on Mini-Protean gels (Biorad, UK). Gels were transferred onto polyvinylidene difluoride (PVDF) in a semidry Trans-Blot Turbo Transfer system (Biorad, UK). The membrane was blocked for at least 1 h at room temperature in blocking buffer (0.1 v/v% tween/PBS supplemented with 5 w/v% milk powder). This was then left to incubate at 4 °C overnight or at room temperature for 1 h with primary antibodies of anti-human HIF-1α (BD, UK) and anti α-tubulin (Abcam, UK) at dilutions of 1:250 and 1:10,000 in blocking buffer, respectively. The membrane was washed and incubated at room temperature for 1 h with a sheep anti-mouse-HRP secondary antibody (GE Healthcare, UK) at a dilution of 1:2500. The membrane was exposed to ECL substrate solution (R&D, UK) for 5 min, and the signal measured using a ChemiDoc-XRS imaging system (Biorad). Signal intensity was analysed using Quantity One software and ImageJ.

### VEGF enzyme linked Immunosorbent assay (ELISA)

Fibroblasts were seeded at a density of 31,250 cells cm^− 2^ in 96 well plates. After attachment overnight, they were rested for 72 h in DMEM + 1 v/v% FCS before treatment with conditioned medium for 24 h. The media were collected to measure VEGF and the cell monolayer was washed in PBS before lysis for a protein assay. 96 well plates were coated in mouse anti-human VEGF capture antibody and blocked in 2 w/v% BSA in PBS before addition of 100 μl of samples and VEGF standards of 0–10,000 pg ml^− 1^ and incubation overnight at 4 °C. The medium was removed, wells washed in 0.05 v/v% tween/PBS, and a detection antibody (biotinylated goat anti-human VEGF) was added for 1 h at room temperature. This was removed and wells incubated with Streptavidin-HRP at a 1:400 dilution for 1 h. After a final wash, 100 μl of substrate solution (R&D) was added per well and incubated for approximately 30 min or until blue coloration was seen in the standard wells. 100 μl of 2 N H_2_SO_4_ was added to stop the reaction and the absorbance read at 450 nm.

### BCA protein assay kit

Cells were lysed in 100 μl of lysis buffer (10 mM Tris pH 8, 1 mM EDTA, 0.2% Triton X-100, protease inhibitor cocktail (Sigma)). A protein assay was performed using the Pierce BCA kit in a 96 well microplate according to the manufacturer’s instructions before measuring the absorbance at 562 nm, and the VEGF production from each sample was normalised to the protein levels.

## Results

### Ion release from the glass particles

**Fig. 1:**
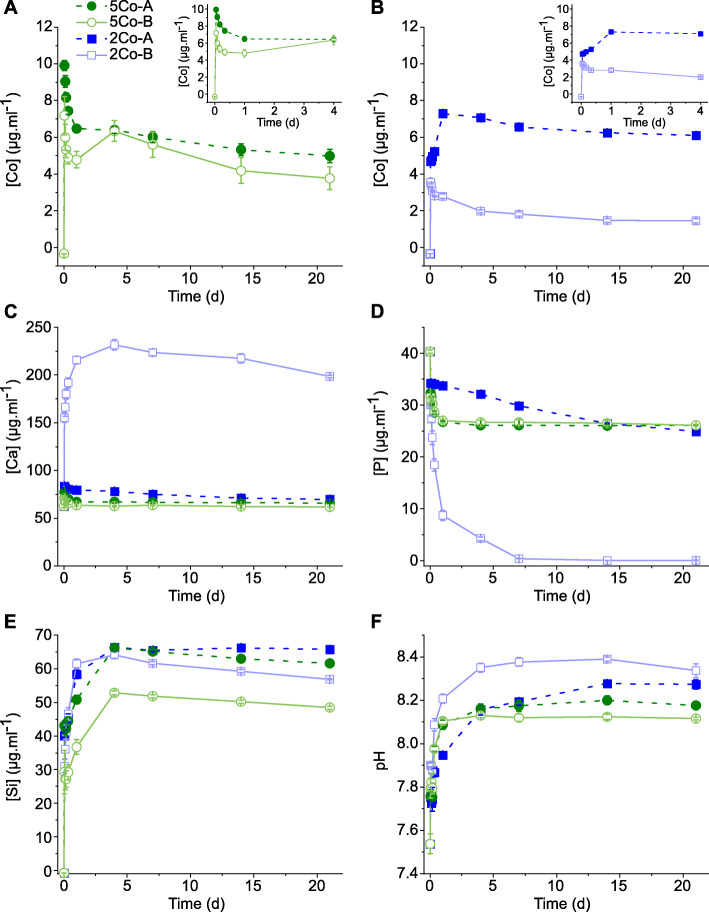
Elemental concentration following dissolution of glasses into SBF over time: **a** Co for 5Co-A and 5Co-B (inset: Co release over the first four days); **b** Co for 2Co-A and 2Co-B (inset: Co release over the first four days); **c** Ca; **d** P; **e** Si; and **f** pH of SBF buffer. Data points are mean ± SD of one independent experiment performed in triplicate

Dissolution and potential of apatite formation for the glasses was investigated in SBF, as SBF is the media used in ISO 23317:2014, the ISO standard approved for detecting apatite formed on a surface of a material. It also enables ion release to be investigated without the interference of proteins. The trend of cobalt ion release from glasses 5Co-A and 5Co-B into SBF was similar (Fig. 1a), with 5Co-B (higher silica content) having a lower cobalt ion release at all time points examined. 2Co-A had a higher cobalt ion release than 2Co-B at all time points, and it was particularly interesting that this glass also had a higher release between 1 and 21 d than 5Co-A and 5Co-B, despite there being less CoO in the glass. Importantly, the amount of Co remained well below 20 μg ml^− 1^, which was previously found to be toxic to cells [32]. Approximately 6 μg ml^− 1^ cobalt was measured in solution at 21 d for 2Co-A, while 5 and 3.5 μg ml^− 1^ were measured for 5Co-A and 5Co-B, respectively. 2Co-B, 5Co-A and 5Co-B all delivered an initial burst release of cobalt ions, over the first 2 h of dissolution, after which there was an overall decrease up to 21 d. 2Co-A was the only glass in which an increase in cobalt was measured between 1 h and 21 d, with the increase being around 30%, although it was approximately constant from 2 d onwards. In comparison, there was a decrease in cobalt from the other three compositions after the first 1 h, with the biggest decrease being for 2Co-B at around 60%, while 5Co-A and 5Co-B each caused a decrease of around 50%, perhaps due to Co incorporation in the surface of the glass.

The calcium concentration slowly decreased over 21 d for 5Co-A, 5Co-B and 2CoA, with a drop of around 10 μg ml^− 1^ between 1 h and 21 d. 2Co-B showed an initial burst release of calcium, which reached a maximum of 232 μg ml^− 1^ by 4 d, which was expected as it was the only composition that contained calcium. After 4 d, the calcium from solution decreased for up to 21 d.

The phosphate concentration in solution decreased rapidly in the first 24 h for 5Co-A and 5Co-B, after which the concentration remained around 26 μg ml^− 1^. For 2Co-A, the decrease in phosphate concentration was slower, and a plateau was not reached within 21 d. Phosphate in the SBF decreased rapidly for 2Co-B between 1 and 24 h, after which the rate of phosphate decrease slowed until the phosphate was completely depleted from solution at 7 d.

The silicon concentration steadily increased between 1 and 4 d. The amount released was similar for 2Co-A, 2Co-B and 5Co-A, with a final concentration between 56 and 65 μg ml^− 1^. The concentration of silicon for 5Co-B was lower than the other compositions at all time points, and measured 48 μg ml^− 1^ at 21 d, which correlated with the higher silica content and NC of this composition.

The release of magnesium and potassium from the glasses is shown in Supplementary information Fig. S1A and B. 5Co-A and 5Co-B are the only compositions to contain K_2_O, and the increase in potassium concentration was similar up to 24 h, at 385 μg ml^− 1^ (5Co-B) and 400 μg ml^− 1^ (5Co-A). An increase in magnesium in the SBF was seen for 5Co-A, 5Co-B and 2Co-A: at 21 d, the magnesium concentration measured 65, 41, and 140 μg ml^− 1^, respectively. For these three compositions, the final concentrations correlated with the amount of MgO in the glass.

As seen in Fig. 1f, for all compositions, the pH of the SBF increased with time, with a plateau reached between 3 and 14 d. The final pH was between 8.1 and 8.4 and correlated to the calculated NC'' of the glasses, with the lowest NC'' glass showing the highest pH.

The dissolution profiles show that the new compositions designed in this study were able to degrade, when incubated in aqueous media, and release therapeutic ions. The decrease in the phosphate content in the SBF for the Ca containing glass (2Co-B) suggests that a calcium phosphate layer may have precipitated on the surface of 2Co-B, but formation of such a layer was unlikely on the other glasses.

### Changes in glass structure

**Fig. 2:**
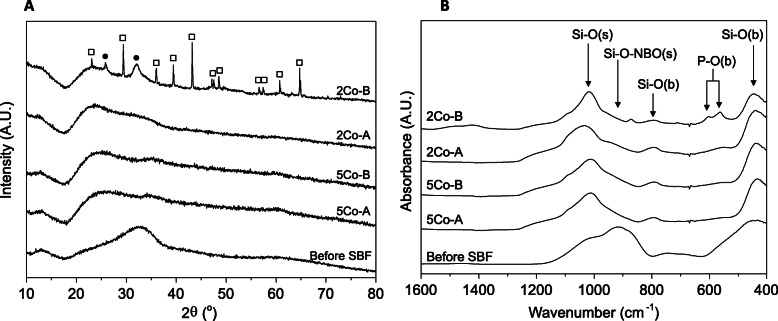
Change in glass structure after incubation in SBF for 21 d: (**a**) XRD and (**b**) FTIR. Filled circles (●) represent peaks relating to apatite and hollow squares (□) represent peaks relating to calcium carbonate. s = stretch, b = bend

To determine whether a crystalline calcium phosphate layer had precipitated on the glass surface, XRD (Fig. 2a) and FTIR (Fig. 2b) were used. All glasses were initially amorphous (Supplementary Information Fig. S2), although 2Co-B showed some small crystalline peaks which could not be identified. All glasses retained their amorphous structure after immersion in SBF, except for 2Co-B which showed broad peaks at 26 and 32° 2θ (Fig. 2a), corresponding to the formation of HCA [82], alongside much sharper peaks representing calcite. Peaks were analysed using the Xpert Highscore Plus database. As the phosphate in the SBF was completely depleted by 7 d of immersion (Fig. 1d), and calcium levels were still high (Fig. 1c), calcium carbonate precipitated. For the other glass compositions, the amorphous halo shifted to the left, perhaps due to glass dissolution changing the glass structure, but there was no evidence of crystallisation.

Prior to incubation in SBF, FTIR spectra for all four compositions were similar. Bands were seen at approximately 1000, 910, 740 and 440 cm^− 1^, which corresponded to the Si-O (stretch), Si-O (stretch) associated with non-bridging oxygens, and two Si-O (bend) vibrations, respectively [83]. After incubation in SBF, the bands associated with Si-O-NBO (stretch) decreased, while the band at 1000 cm^− 1^ related to Si-O (stretch) became more prominent (Fig. 2b). The wide band at approximately 740 cm^− 1^ shifted to around 790 cm^− 1^ after incubation and corresponded to the Si-O (bending) vibrations. For 2Co-B, bands at 560 and 600 cm^− 1^ indicated the presence of crystalline orthophosphate, such as HCA, which was confirmed by XRD (Fig. 2a) [82].

### Changes at the glass surface

**Fig. 3:**
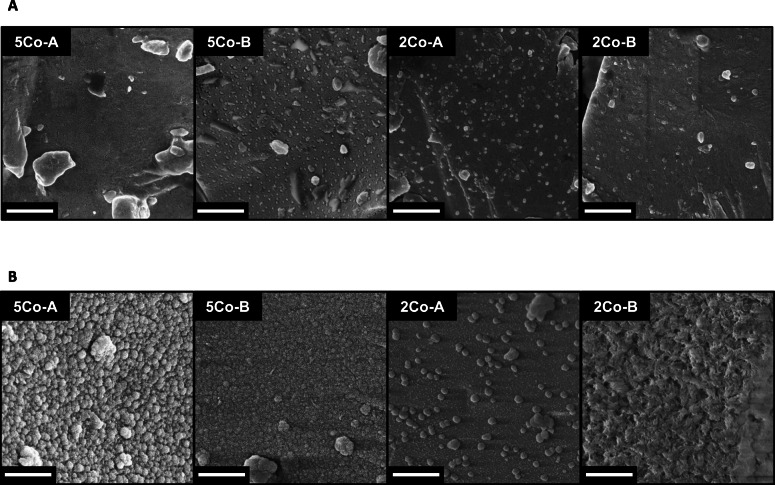
SEM images showing changes in glass surface morphology of glass particles (**a**) before and (**b**) after incubation in SBF for 21 d. Scale bar is 1 μm

The decrease in cobalt, calcium and phosphate in SBF over time suggested that there may have been deposition, although no calcium phosphate deposition was detected using XRD and FTIR for glasses 2Co-A, 5Co-B and 5Co-A. SEM images (Fig. 3) showed that the glass particles were free of precipitate before incubation in SBF. After 21 d in SBF, a precipitated layer could be seen in all four glass compositions. 5Co-A and 5Co-B had a homogenous layer of precipitate, with the layer on 5Co-A appearing to be more abundant. Little change was observed for the surface of 2Co-A, although some isolated precipitates were visible. In contrast, an angular, precipitated layer was seen all over the glass particles of 2Co-B, which was likely to be a mix of calcium carbonate and calcium phosphate. The faceted appearance of many of the crystallites was typical of calcite [84].

To further characterise the formation of the surface layers, sections were cut, by FIB, from the top of the glass particles, and TEM-EDS was used to detect the elemental composition of these layers without interference from the elements in the underlying glass. Glass 5Co-A and 2Co-A were of particular interest because despite 5Co-A having a higher amount of cobalt in the glass composition, the cobalt concentration in SBF at 21 d was lower than that released from 2Co-A. For this reason, these two compositions were analysed by TEM-EDS.

**Fig. 4:**
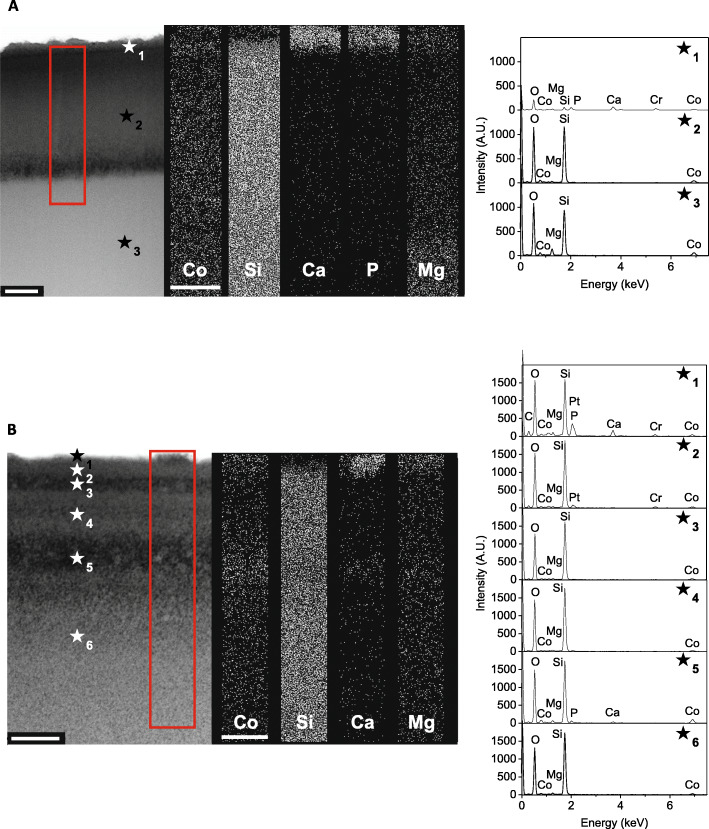
TEM-EDS analysis of the surface cross-sections of (**a**) 5Co-A and (**b**) 2Co-A particles after incubation in SBF for 21 d, and with individual spectra taken at multiple locations marked by a ★. Sections were cut perpendicular to the glass surface by FIB. Scale bar is 250 nm

Figure 4a and b show the composition at the surface of the glass particles for 5Co-A and 2Co-A respectively, after the glass was incubated for 21 d in SBF buffer. For both glass compositions, distinct reaction layers could be seen. The 5Co-A particles showed 3 clear layers. The first was a calcium phosphate layer at the top surface of the particle (Fig. 4a - ★_1_), into which cobalt and magnesium precipitated and where silica was depleted. There was a lower amount of magnesium, in the second layer (Fig. 4a - ★_2_), while the third layer had a similar composition, although it contained more magnesium (Fig. 4a - ★_3_). Calcium and phosphate were not detected when measuring individual spectra of these two layers, which suggested that these were present solely on the surface of the glass.

For 2Co-A there were several reaction layers, which could not be easily distinguished from each other. Small amounts of calcium and phosphorous were detected in the uppermost layer (Fig. 4b ★_1_), by EDX, which corresponded to the small areas of precipitate seen under SEM (Fig. 3b). Both cobalt and magnesium were detected throughout the layers ★_1_ to ★_6_.

Prior to immersion in SBF, no reaction layers were observed at the surface of the particles for either 5Co-A or 2Co-A (Fig. S3). The samples prior to incubation in SBF were particularly beam sensitive, which led to some brighter areas being observed in 2Co-A, but this was not expected to affect the measurement of the elemental composition.

### Electrospinning of composites

**Fig. 5:**
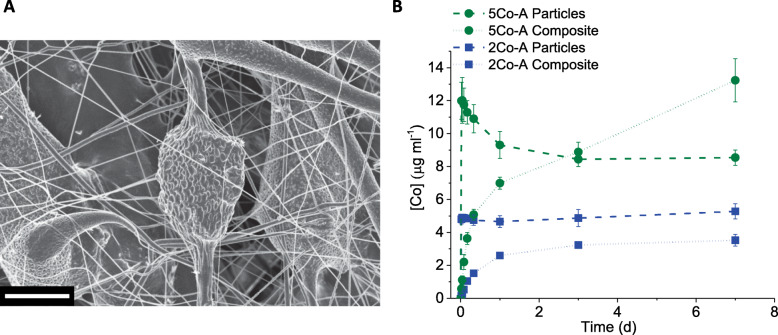
**a** SEM image of a typical electrospun composite. Scale bar is 20 μm. **b** Co ion release from glass particles and composite in DMEM cell culture media at 0.5 h, 1 h, 2 h, 4 h, 8 h, 24 h, 3 d and 7 d. Data shown is mean ± SD of one independent experiment performed in triplicate

Electrospun composites were produced with two glass compositions, 5Co-A and 2Co-A, and a loading of 30 wt% of glass particles was achieved. Preliminary experiments showed that with lower glass loadings, fibres were not formed, and the fibres did not hold together with higher glass loadings. After electrospinning, two types of fibres were observed; smaller fibres that had a uniform diameter of less than 1 μm, and larger fibres ranging from 1 to 75 μm (Fig. 5a and Fig. S4). The smaller fibres were composed solely of PCL, while the larger fibres contained glass particles, and therefore varied in diameter along their length (Fig. 5a).

The ion release from these composites was measured in DMEM cell culture media (without cells) and compared to the dissolution of glass particles alone. After 1 h, there was a burst release of cobalt ions from the glass particles, with the composite showing a more controlled increase. For the 5Co-A composite, the cobalt ion release matched that from the glass particles at 3 d at around 8 μg ml^− 1^ and continued to increase to 13 μg ml^− 1^ at 7 d, while the ion release from the particles plateaued at around 8 μg ml^− 1^ by 3 d. For the 2Co-A composite, the cobalt concentration increased until a plateau was reached after 3 d at about 3 μg ml^− 1^; in contrast the ion release from the glass particles plateaued at around 5 μg ml^− 1^. This followed a similar trend to the ion release in SBF buffer, although the cobalt concentration in DMEM is higher for 5Co-A. This was likely to be a result of a smaller calcium phosphate layer formed in DMEM compared to SBF, as SBF is supersaturated in calcium and phosphate. As a result, there was less precipitation of cobalt and an increased concentration in solution.

Incorporating glass particles into an electrospun composite meant these particles were not released during the duration of the study. The presence of the PCL had an additional benefit of controlling the ion release over the first 24 h of incubation, avoiding the burst ion release which was seen in the glass particles.

### In vitro response to composites

**Fig. 6:**
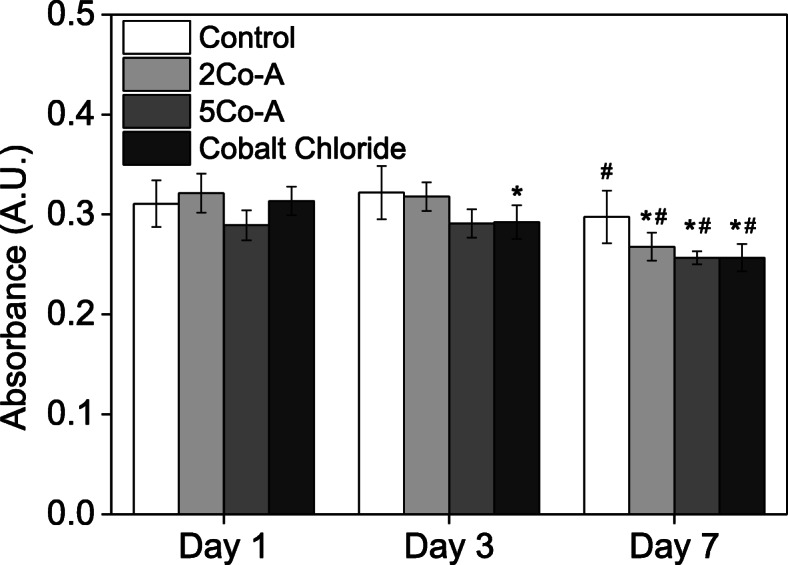
AlamarBlue reading of cells in contact with control (DMEM) media, conditioned media from glass composites, and DMEM containing 100 μM CoCl_2_. Data shown is mean ± SD of three independent experiments. Two way ANOVA conducted with Tukey test with * *p* < 0.05 compared to Day 1 and # *p* < 0.05 compared to Day 3

Fibroblasts play a crucial role during wound healing through the expression of growth factors, synthesising the extracellular matrix and by mediating contraction to close the wound. For this reason, fibroblasts were exposed to conditioned media from the glass composites and the effect on proliferation, HIF-1α stabilisation and production of VEGF were measured. Figure 6 shows the metabolic activity of primary fibroblasts when exposed to conditioned media for up to 7 d. The fibroblasts maintained their metabolic activity when exposed to the conditioned media and 100 μM cobalt chloride for up to 3 d, after which there was a significant decrease in metabolic activity. This demonstrated there was very little detrimental effect of the composites on the metabolic activity of primary fibroblasts for up to 3 d, in the conditions used for this study. However, a decrease was seen between 3 and 7 d, which is in line with reports that have shown a detrimental effect on metabolic activity of fibroblast after long term exposure to CoCl_2_ [85].

**Fig. 7:**
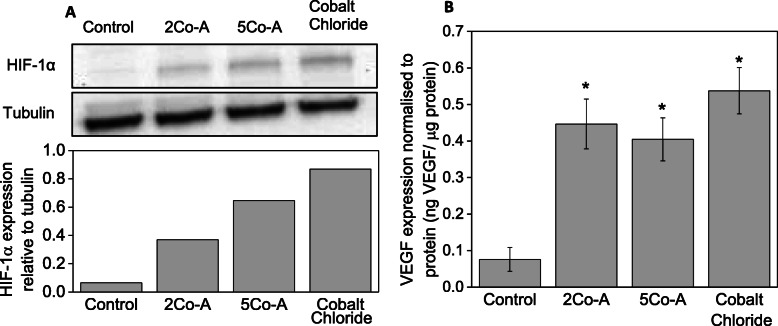
**a** A typical western blot showing increased expression of HIF-1α with conditioned media and representative western blot quantification (representative images shown from a total of three independent experiments); (**b**) VEGF expression of fibroblasts relative to protein: data shown are mean ± SD of three independent experiments. One way ANOVA performed with * *p* < 0.05

Increased HIF-1α stabilisation when fibroblasts were exposed to conditioned media and cobalt chloride is shown in Fig. 7 a. HIF-1α stabilisation increased in the order 2Co-A, 5Co-A and cobalt chloride. Downstream effects of stabilising HIF-1α include the increased expression of proteins such as VEGF [32]. When exposed to conditioned media or CoCl_2_, the expression of VEGF by fibroblasts was found to be significantly higher than DMEM controls (Fig. 7). There was no significant difference in the amount of VEGF produced after exposing fibroblasts to 100 μM CoCl_2_ medium or the composite-conditioned media.

## Discussion

The aim of this study was to produce cobalt doped glass compositions, which released cobalt ions to activate the HIF pathway by stabilising HIF-1α, without forming an HCA layer on their surface after incubation in SBF. The role of magnesium in glass compositions was investigated to suppress HCA layer formation but maintain low network connectivity for glass dissolution and cobalt release.

The glass that contained calcium in the composition caused calcium phosphate precipitation from the SBF (Fig. 1) and HCA formation (Fig. 2). There was no evidence for crystalline phase deposition on the other glasses from XRD (Fig. 2), but a thin layer, which could be an amorphous layer, was seen by SEM on all glasses except 2Co-A (Fig. 3), which only showed some isolated spherical deposits. The slight decrease in Co content of the SBF suggests the thin layer could contain Co.

FIB sections (Fig. 4) allowed the measurement of the elemental composition with good spatial accuracy and without the composition of the underlying glass interfering with the measurement (which would have occurred with SEM-EDS). TEM and EDS showed the formation of a mixed magnesium and cobalt substituted calcium phosphate at the surface of both 5Co-A and 2Co-A particles, which was less than 100 nm in thickness. However, as neither of these glasses contained calcium or phosphate, it is thought that the presence of the glass particles, in combination with the increased local pH caused the particles to act as nucleation sites for calcium phosphate to precipitate from the SBF [12, 86]. The calcium phosphate layer formed for 5Co-A was much larger and more distinct, allowing it to be clearly seen in the TEM images, while for 2Co-A it was concentrated in small areas as seen by SEM (Fig. 3).

For both compositions, sodium (or potassium) was not detected in the glass after 21 d in SBF. Fredholm et al. [87], showed that for a strontium substituted glass, sodium was depleted from the top 6 μm after incubation for 1 week in SBF. The sections prepared here by FIB and observed under the TEM, were approximately 5 μm in depth and it is likely that after incubating glasses for 21 d, the sodium was depleted from a much larger area than this. It was anticipated that the large area of sodium depletion was due to the size and monovalent nature of the sodium ion, which means it is more mobile and able to travel through the glass into the aqueous solution more easily.

The cobalt concentration in SBF at 21 d was lower for 5Co-A compared to 2Co-A, despite there being a higher amount of CoO in 5Co-A, which was likely to be due to 5Co-A having a higher NC’. Through the use of TEM-EDS, a layer of cobalt and magnesium substituted calcium phosphate was observed on the surface of 5Co-A particles, while for 2Co-A this was confined to small regions of precipitate (Fig. 3b). The drop in cobalt concentration following the burst release after 1 h for 5Co-A (Fig. 2a) was likely to be a result of a larger amount of calcium phosphate precipitating on the glass, into which cobalt was incorporated.

2Co-A did not show the same trend of cobalt ion release as the other glasses studied. There was an initial burst release of ions after 1 h, with the cobalt concentration continuing to increase for up to 21 d. There was less cobalt substituted calcium phosphate nucleating on this glass, and so the cobalt was released into the SBF rather than being precipitated back onto the glass surface. This could have been due to the relatively high amount of magnesium in the glass composition which has previously been shown to impede the formation of a calcium phosphate layer [41, 88]. The TEM images also suggest that Co and Mg were present throughout the reaction layers of the glass, indicating that they play a role in the silica-rich layer, as previously seen in glasses designed for nuclear waste immobilisation [89].

It has previously been reported that cobalt can substitute into hydroxyapatite [90] and into a calcium phosphate layer on bioactive glasses [40]. However, this study showed for the first time the decrease in cobalt ions from solution following the initial burst release of ions. This was the case for three out of the four glass compositions studied (5Co-A, 5Co-B and 2Co-B), and corresponded to the formation of cobalt and magnesium substituted calcium phosphate. In addition to the cobalt and magnesium calcium phosphate layer, TEM and EDS also revealed a number of surface layers, particularly for 2Co-A which had multiple layers of the same elemental composition, which appeared to have a different porosity. These layers may have been partly due to the formation of a silica gel layer, which has been widely reported to form on the surface of glass when placed in aqueous media [91]. This silica gel layer is initially formed as a hydrated porous layer before repolymerising on the glass surface [92]. For bioactive glasses, this layer has been shown to have a thickness of around 2–3 μm after 1 h incubation in TRIS buffer [92], or greater than 30 μm after 7 d in SBF buffer [87]. In this study, layers closest to the surface were imaged and the edge of the whole silica gel layer was not observed.

The layers observed in 2Co-A were much like those reported by Cailleteau et al. [91], who showed that an initial porous silica gel layer was made more dense by repolymerisation. Magnesium ions in solution can change the structure of the silica gel layer, by hindering the condensation of the silica gel layer, which results in a layer that is thicker and more porous [93]. Since 2Co-A contained a high molar percentage of MgO, high amounts of magnesium ions were released into the buffer upon glass degradation. This may have hindered the repolymerisation of the silica network, and led to an incomplete densification of the silica gel layer after 21 d. Although two porous layers were seen between three denser layers of the same composition (Fig. 4b), this observation was not in alignment with other reports demonstrating the denser silica layer was formed at the top surface of the glass [91]. Interestingly, the second porous layer (Fig. 4b, ★_5_) seemed to be concentrated in cobalt, magnesium, calcium and phosphorous. More investigations are required to understand the formation and densification of the silica gel layer in complex bioactive glasses when incubated in buffers with ion concentrations similar to physiological values.

When considering bioactive glasses for wound healing applications, an important consideration is the format in which the glass will be delivered to the clinician and patient. The dressing format would need to prevent the release of small glass particles, which could be a source of irritation for the patient and would also be difficult to wash out of a wound during dressing changes, without compromising the ability of the glass to release ions that could promote wound healing. Electrospun composites were produced from two glass compositions, 5Co-A and 2Co-A, and in this format, the glass particles were held in place for up to 1 week, but ions were still released from the glass. The electrospun composites had fibres with diameters less than 100 μm and showed a sustained release of cobalt ions over the first 24 h incubation in cell culture media, when compared to the glass particles, in which a typical burst release of ions was seen.

Cobalt ions are only therapeutic within a certain concentration range, above which they are toxic, and this range is dependent on the cell type and the cobalt ion delivery system under investigation. Wu et al. [39] found mesoporous sol-gel bioactive glass scaffolds, which released approximately 17 μg ml^− 1^ cobalt after 7 d in cell culture media, could still support cell growth on their surface, without a significant reduction in cell viability compared to a control in human bone marrow stromal cells. In contrast, glasses that released 20 μg ml^− 1^ of cobalt showed a significant reduction in viability. In hMSC culture, Azevedo et al. [32] found that the therapeutic range of cobalt was between approximately 2.2 and 10.6 μg ml^− 1^; and at higher concentrations, a significant reduction in proliferation was observed. The glasses and composites developed in this study had a maximum cobalt release of 14.5 μg ml^− 1^ and so were not expected to have a toxic effect. In vitro cell studies with primary human fibroblasts confirmed that the composites containing particles of these new glass compositions did not have a toxic effect for up to 3 d, and were able to activate the HIF pathway by stabilising HIF-1α.

In order to examine the effects of the ions on proliferation, HIF-1α stabilisation and production of VEGF, fibroblasts were exposed to media that had been conditioned with the glass composite for 24 h. The metabolic activity of cells was similar to those cultured in unconditioned growth media and cobalt chloride controls (Fig. 6). A decrease in metabolic activity was observed after 3 d and this correlates with previous findings in the literature that showed a reduction in metabolic activity after long term exposure to CoCl_2_ [85].

Cobalt was incorporated into these glass compositions to mimic hypoxia by activating the HIF-1 pathway. The amount of HIF-1α increased when fibroblasts were exposed to conditioned media from both 5Co-A and 2Co-A composites as well as the CoCl_2_ control. This showed that the composite stabilised HIF-1α, and this was expected to increase production of proteins required for angiogenesis. There was a clear trend that the measured HIF-1α was higher for 5Co-A compared to 2Co-A. More cobalt was released from the 5Co-A compositions compared to the 2Co-A at 24 h (Fig. 1a-b and Fig. 5b), which is likely to be the reason for the increased HIF-1α stabilisation.

Finally, there was a significant increase in VEGF expression in fibroblasts when using both conditioned media from the scaffolds and CoCl_2_, compared to growth medium (Fig. 7). However, there was no significant difference in the amount of VEGF produced by fibroblasts exposed to both the 5Co-A and 2Co-A composites, which suggested that both compositions would be suitable in promoting angiogenesis. It is challenging to dissociate the effects of individual ions. Bioactive glasses dissolution products (without potassium, cobalt or magnesium) have previously been shown to directly promote VEGF expression from fibroblasts [94, 95]. Here, it was possible that other ions released from the scaffolds, such as potassium and magnesium, might also have affected protein expression by the cells, as they were released by the glasses (Fig. S1). A deficiency in magnesium can affect platelet activity [96] and inhibit angiogenesis [97], while increased magnesium promoted phenotype switching of macrophages in vitro [98] and proliferation of microvascular cells [97]. However, studies with human umbilical vein endothelial cells (HUVECS) showed increased magnesium concentration did not increase VEGF expression under normoxia or hypoxia, so it is unlikely to have an effect here [99]. Figure 7 indicates that the combination of ions from the scaffolds did not inhibit VEGF production compared to CoCl_2_.

## Conclusions

Cobalt containing silicate glass compositions were designed to avoid the formation of a HCA layer following incubation in SBF buffer, and yet retain the ability to allow cobalt ions to be released. Two series of glass compositions were designed containing either 5 mol% or 2 mol% CoO and the amount of cobalt released into solution was found to not only be related to the initial amount of cobalt in the glass, but also network connectivity of the glass and the surface layer that formed during dissolution.

TEM-EDS analysis of FIB cross-sections of the surface reactions layers revealed the presence of a cobalt and magnesium substituted calcium phosphate, which caused a decrease in Co concentrations in SBF. Several layers were seen through the glass particles after SBF incubation, which were dependent on the glass composition which affected how the silica-rich gel layer repolymerised during dissolution. The 2Co-A (50 mol% SiO_2_, 24 mol% Na_2_O, 24 mol% MgO and 2 mol% CoO) composition showed negligible calcium phosphate deposition and sustained Co release.

Electrospinning PCL/glass composites prevented a burst release of ions from the glass particles during dissolution. The metabolic activity of primary fibroblasts was not significantly reduced for up to 3 d when the cells were in contact with conditioned media from the composites. The conditioned media from both composites stabilised HIF-1α and significantly increased the expression of VEGF in fibroblasts.

In summary, due to their combination of promoting VEGF expression, stabilising HIF-1α, while not forming an apatite layer, glasses based on the novel composition 50 mol% SiO_2_, 24 mol% Na_2_O, 24 mol% MgO and 2 mol% CoO, and their electrospun composites, have potential to promote angiogenesis and these materials could be beneficial in wound healing applications.

## Supplementary Information

**Additional file 1: Figure S1.** (A) Potassium and (B) Magnesium ion release from all glasses in SBF after 1, 2, 4, 6, 24 h, 3, 7, 14 and 21 d. **Figure S2.** XRD patterns of all four glass compositions before incubation in SBF buffer. **Figure S3.** TEM-EDS analysis of glass structure of (A) 5Co-A and (B) 2Co-A before incubation in SBF buffer. Scale bar is 200 nm. **Figure S4.** Representative SEM image of the 2Co-A electrospun composite at low magnification showing the wide variety of fibre diameters. Scale bar is 100 μm.

## Data Availability

Raw data can be obtained from rdm-enquiries@imperial.ac.uk

## References

[1] Jones JR (2013). Review of bioactive glass: from Hench to hybrids. Acta Biomater..

[2] Rahaman MN, Day DE, Bal BS, Fu Q, Jung SB, Bonewald LF (2011). Bioactive glass in tissue engineering. Acta Biomater.

[3] Hench LL (2015). Opening paper 2015- some comments on bioglass: four eras of discovery and development. Biomedical Glasses.

[4] Jones JR, Brauer DS, Hupa L, Greenspan DC (2016). Bioglass and bioactive glasses and their impact on healthcare. Int J Appl Glas Sci.

[5] Hench LL, Splinter RJ, Allen WC, Greenlee TK (1971). Bonding mechanisms at the interface of ceramic prosthetic materials. J Biomed Mater Res Symp.

[6] Greenspan DC (2019). Bioglass at 50 – a look at Larry Hench’s legacy and bioactive materials. Biomedical Glasses..

[7] Xynos ID, Edgar AJ, Buttery LDK, Hench LL, Polak JM (2001). Gene-expression profiling of human osteoblasts following treatment with the ionic products of bioglass (R) 45S5 dissolution. J Biomed Mater Res.

[8] Oonishi H, Hench LL, Wilson J, Sugihara F, Tsuji E, Kushitani S (1999). Comparative bone growth behavior in granules of bioceramic materials of various sizes. J Biomed Mater Res.

[9] Oonishi H, Hench LL, Wilson J, Sugihara F, Tsuji E, Matsuura M (2000). Quantitative comparison of bone growth behavior in granules of bioglass (R), A-W glass-ceramic, and hydroxyapatite. J Biomed Mater Res.

[10] Kokubo T, Takadama H (2006). How useful is SBF in predicting in vivo bone bioactivity?. Biomaterials..

[11] Hench LL, Clark DE (1978). Physical chemistry of glass surfaces. J Non-Cryst Solids.

[12] Bohner M, Lemaitre J (2009). Can bioactivity be tested in vitro with SBF solution?. Biomaterials..

[13] Hoppe A, Guldal NS, Boccaccini AR (2011). A review of the biological response to ionic dissolution products from bioactive glasses and glass-ceramics. Biomaterials..

[14] Baino F, Novajra G, Miguez-Pacheco V, Boccaccini AR, Vitale-Brovarone C (2016). Bioactive glasses: special applications outside the skeletal system. J Non-Cryst Solids.

[15] Kargozar S, Hamzehlou S, Baino F (2019). Can bioactive glasses be useful to accelerate the healing of epithelial tissues?. Mater Sci Eng C..

[16] Lin C, Mao C, Zhang JJ, Li YL, Chen XF. Healing effect of bioactive glass ointment on full-thickness skin wounds. Biomed Mater. 2012;7(4). Article number: 045017. 10.1088/1748-6041/7/4/045017 22736113

[17] Zhao SC, Li L, Wang H, Zhang YD, Cheng XG, Zhou N (2015). Wound dressings composed of copper-doped borate bioactive glass microfibers stimulate angiogenesis and heal full-thickness skin defects in a rodent model. Biomaterials..

[18] Lin YN, Brown RF, Jung SB, Day DE (2014). Angiogenic effects of borate glass microfibers in a rodent model. J Biomed Mater Res Part A..

[19] Day R, Boccaccini AR, Roether JA, Surey S, Forbes A, Hench LL (2002). The effect of Bioglass((R)) on epithelial cell and fibroblast proliferation and incorporation into a PGA matrix. Gastroenterology.

[20] Leu A, Leach JK (2008). Proangiogenic potential of a collagen/bioactive glass substrate. Pharm Res.

[21] Liu X, Rahaman MN, Day DE (2014). In vitro degradation and conversion of melt-derived microfibrous borate (13-93B3) bioactive glass doped with metal ions. J Am Ceram Soc.

[22] Ostomel TA, Shi QH, Tsung CK, Liang HJ, Stucky GD (2006). Spherical bioactive glass with enhanced rates of hydroxyapatite deposition and hemostatic activity. Small..

[23] Tokoro S, Satoh T, Okubo Y, Igawa K, Yokozeki H (2009). Latent dystrophic subcutaneous calcification in patients with chronic venous insufficiency. Acta Derm Venereol.

[24] Wollina U, Hasenohrl K, Kostler E, Schonlebe J, Heinig B, Haroske G (2009). Dystrophic calcification in chronic leg ulcers - a Clinicopathologic study. Dermatol Surg.

[25] Milas M, Bush RL, Lin P, Brown K, Mackay G, Lumsden A (2003). Calciphylaxis and nonhealing wounds: the role of the vascular surgeon in a multidisciplinary treatment. J Vasc Surg.

[26] Mace KA, Yu DH, Paydar KZ, Boudreau N, Young DM (2007). Sustained expression of Hif-1 alpha in the diabetic environment promotes angiogenesis and cutaneous wound repair. Wound Repair Regen.

[27] Botusan IR, Sunkari VG, Savu O, Catrina AI, Grunler J, Lindberg S (2008). Stabilization of HIF-1 alpha is critical to improve wound healing in diabetic mice. PNAS..

[28] Shi QY, Luo X, Huang ZQ, Midgley AC, Wang B, Liu RH (2019). Cobalt-mediated multi-functional dressings promote bacteria-infected wound healing. Acta Biomater.

[29] Yuan Y, Hilliard G, Ferguson T, Millhorn DE (2003). Cobalt inhibits the interaction between hypoxia-inducible factor-alpha and von Hippel-Lindau protein by direct binding to hypoxia-inducible factor-alpha. J Biol Chem.

[30] Lee JW, Bae SH, Jeong JW, Kim SH, Kim KW (2004). Hypoxia-inducible factor (HIF-1) alpha: its protein stability and biological functions. Experimental Mol Med.

[31] Dery MAC, Michaud MD, Richard DE (2005). Hypoxia-inducible factor 1: regulation by hypoxic and non-hypoxic activators. Int J Biochem Cell Biol.

[32] Azevedo MM, Tsigkou O, Nair R, Jones JR, Jell G, Stevens MM (2015). Hypoxia inducible factor-stabilizing bioactive glasses for directing mesenchymal stem cell behavior. Tissue Eng Part A.

[33] Namiki A, Brogi E, Kearney M, Kim EA, Wu TG, Couffinhal T (1995). Hypoxia induces vascular endothelial growth factor in cultured human endothelial cells. J Biol Chem.

[34] Hoppe A, Brandl A, Bleiziffer O, Arkudas A, Horch RE, Jokic B (2015). In vitro cell response to co-containing 1393 bioactive glass. Mater Sci Eng, C.

[35] Trompezinski S, Pernet I, Mayoux C, Schmitt D, Viac J (2000). Transforming growth factor-beta 1 and ultraviolet A1 radiation increase production of vascular endothelial growth factor but not endothelin-1 in human dermal fibroblasts. Br J Dermatol.

[36] Zhao SC, Wang H, Zhang YD, Huang WH, Rahaman MN, Liu ZT (2015). Copper-doped borosilicate bioactive glass scaffolds with improved angiogenic and osteogenic capacity for repairing osseous defectse. Acta Biomater.

[37] Xie ZW, Paras CB, Weng H, Punnakitikashem P, Su LC, Vu K (2013). Dual growth factor releasing multi-functional nanofibers for wound healing. Acta Biomater.

[38] Leach JK, Kaigler D, Wang Z, Krebsbach PH, Mooney DJ (2006). Coating of VEGF-releasing scaffolds with bioactive glass for angiogenesis and bone regeneration. Biomaterials..

[39] Wu C, Zhou Y, Fan W, Han P, Chang J, Yuen J (2012). Hypoxia-mimicking mesoporous bioactive glass scaffolds with controllable cobalt ion release for bone tissue engineering. Biomaterials..

[40] Hoppe A, Jokic B, Janackovic D, Fey T, Grei P, Romeis S (2014). Cobalt-releasing 1393 bioactive glass-derived scaffolds for bone tissue engineering applications. ACS Appl Mater Interf.

[41] Azevedo MM, Jell G, O’Donnell MD, Law RV, Hill RG, Stevens MM (2010). Synthesis and characterization of hypoxia-mimicking bioactive glasses for skeletal regeneration. J Mater Chem.

[42] Smith JM, Martin RA, Cuello GJ, Newport RJ (2013). Structural characterisation of hypoxia-mimicking bioactive glasses. J Mat Chem B.

[43] Ciceo RL, Todea M, Dudric R, Buhai A, Simon V (2018). Structural effect of cobalt ions added to a borophosphate-based glass system. J Non-Cryst Solids.

[44] Raja FNS, Worthington T, Isaacs MA, Chungong LF, Burke B, Addison O (2019). The antimicrobial efficacy of hypoxia mimicking cobalt oxide doped phosphate-based glasses against clinically relevant gram positive, gram negative bacteria and a fungal strain. Acs Biomaterials Science & Engineering.

[45] Deng ZW, Lin BC, Jiang ZH, Huang WH, Li JS, Zeng XQ (2019). Hypoxia-mimicking cobalt-doped borosilicate bioactive glass scaffolds with enhanced angiogenic and osteogenic capacity for bone regeneration. Int J Biol Sci.

[46] Barrioni BR, de Laia AGS, Valverde TM, Martins TMD, Caliari MV, de Sa MA (2018). Evaluation of in vitro and in vivo biocompatibility and structure of cobalt releasing sol-gel bioactive glass. Ceram Int.

[47] Barrioni BR, Norris E, Jones JR, Pereira MD (2018). The influence of cobalt incorporation and cobalt precursor selection on the structure and bioactivity of sol-gel-derived bioactive glass. J Sol-Gel Sci Technol.

[48] Dziadek M, Zagrajczuk B, Menaszek E, Dziadek K, Cholewa-Kowalska K (2018). A simple way of modulating in vitro angiogenic response using cu and co-doped bioactive glasses. Mater Lett.

[49] de Laia AGS, Barrioni BR, Valverde TM, de Goes AM, de Sa MA, Pereira MD (2020). Therapeutic cobalt ion incorporated in poly (vinyl alcohol)/bioactive glass scaffolds for tissue engineering. J Mater Sci.

[50] Goh Y-F, Alshemary AZ, Akram M, Kadir MRA, Hussain R (2013). In vitro study of nano-sized zinc doped bioactive glass. Mater Chem Phys.

[51] Poologasundarampillai G, Lee PD, Lam C, Kourkouta AM, Jones JR (2016). Compressive strength of bioactive sol-gel glass foam scaffolds. Int J Appl Glas Sci.

[52] Nommeots-Nomm A, Labbaf S, Devlin A, Todd N, Geng H, Solanki AK (2017). Highly degradable porous melt-derived bioactive glass foam scaffolds for bone regeneration. Acta Biomater.

[53] Nommeots-Nomm A, Lee PD, Jones JR (2018). Direct ink writing of highly bioactive glasses. J Eur Ceram Soc.

[54] Fu Q, Saiz E, Rahaman MN, Tomsia AP (2011). Bioactive glass scaffolds for bone tissue engineering: state of the art and future perspectives. Mater Sci Eng, C..

[55] Kim H-W, Kim H-E, Knowles JC (2006). Production and potential of bioactive glass nanofibers as a next-generation biomaterial. Adv Funct Mater.

[56] Quintero F, Pou J, Comesana R, Lusquinos F, Riveiro A, Mann AB (2009). Laser spinning of bioactive glass Nanofibers. Adv Funct Mater.

[57] Poh PSP, Hutmacher DW, Holzapfel BM, Solanki AK, Stevens MM, Woodruff MA (2016). In vitro and in vivo bone formation potential of surface calcium phosphate-coated polycaprolactone and polycaprolactone/bioactive glass composite scaffolds. Acta Biomater.

[58] Paxton NC, Ren JY, Ainsworth MJ, Solanki AK, Jones JR, Allenby MC (2019). Rheological characterization of biomaterials directs additive manufacturing of strontium-substituted bioactive glass/Polycaprolactone microfibers. Macromol Rapid Commun.

[59] Rezwan K, Chen QZ, Blaker JJ, Boccaccini AR (2006). Biodegradable and bioactive porous polymer/inorganic composite scaffolds for bone tissue engineering. Biomaterials..

[60] Poologasundarampillai G, Wang D, Li S, Nakamura J, Bradley R, Lee PD (2014). Cotton-wool-like bioactive glasses for bone regeneration. Acta Biomater.

[61] Poologasundarampillai G, Yu BB, Jones JR, Kasuga T (2011). Electrospun silica/PLLA hybrid materials for skeletal regeneration. Soft Matter.

[62] Yoshimoto H, Shin YM, Terai H, Vacanti JP (2003). A biodegradable nanofiber scaffold by electrospinning and its potential for bone tissue engineering. Biomaterials..

[63] McCullen SD, Autefage H, Callanan A, Gentleman E, Stevens MM (2012). Anisotropic fibrous scaffolds for articular cartilage regeneration. Tissue Eng Part A..

[64] Kim HW, Lee HH, Knowles JC (2006). Electrospinning biomedical nanocomposite fibers of hydroxyapaite/poly (lactic acid) for bone regeneration. J Biomed Mater Res Part A..

[65] Peng F, Yu XH, Wei M (2011). In vitro cell performance on hydroxyapatite particles/poly(L-lactic acid) nanofibrous scaffolds with an excellent particle along nanofiber orientation. Acta Biomater.

[66] Obata A, Ozasa H, Kasuga T, Jones JR (2013). Cotton wool-like poly (lactic acid)/vaterite composite scaffolds releasing soluble silica for bone tissue engineering. J Mater Sci - Mater Med.

[67] Schneider OD, Weber F, Brunner TJ, Loher S, Ehrbar M, Schmidlin PR (2009). In vivo and in vitro evaluation of flexible, cottonwool-like nanocomposites as bone substitute material for complex defects. Acta Biomater.

[68] Ren JY, Blackwood KA, Doustgani A, Poh PP, Steck R, Stevens MM (2014). Melt-electrospun polycaprolactone strontium-substituted bioactive glass scaffolds for bone regeneration. J Biomed Mater Res Part A..

[69] Kouhi M, Morshed M, Varshosaz J, Fathi MH (2013). Poly (epsilon-caprolactone) incorporated bioactive glass nanoparticles and simvastatin nanocomposite nanofibers: preparation, characterization and in vitro drug release for bone regeneration applications. Chem Eng J.

[70] Moura D, Souza MT, Liverani L, Rella G, Luz GM, Mano JF (2017). Development of a bioactive glass-polymer composite for wound healing applications. Mater Sci Eng, C..

[71] Zahedi P, Rezaeian I, Ranaei-Siadat SO, Jafari SH, Supaphol P (2010). A review on wound dressings with an emphasis on electrospun nanofibrous polymeric bandages. Polym Adv Technol.

[72] Ma ZJ, Ji HJ, Tan DZ, Teng Y, Dong GP, Zhou JJ (2011). Silver nanoparticles decorated, flexible SiO2 nanofibers with long-term antibacterial effect as reusable wound cover. Colloid Surf A-Physicochem Eng Asp.

[73] Li XF, Liu Y, Zhang J, You RC, Qu J, Li MZ (2017). Functionalized silk fibroin dressing with topical bioactive insulin release for accelerated chronic wound healing. Mater Sci Eng, C..

[74] Lai HJ, Kuan CH, Wu HC, Tsai JC, Chen TM, Hsieh DJ (2014). Tailored design of electrospun composite nanofibers with staged release of multiple angiogenic growth factors for chronic wound healing. Acta Biomater.

[75] Gillette B, Criscitelli T, Howell R, Woods J, Acerra M, Gorenstein S (2019). Regenerative wound surgery: practical application of regenerative medicine in the OR. AORN J.

[76] Gillette RL, Swaim SF, Sartin EA, Bradley DM, Coolman SL (2001). Effects of a bioactive glass on healing of closed skin wounds in dogs. Am J Vet Res.

[77] Watts SJ, Hill RG, O'Donnell MD, Law RV (2010). Influence of magnesia on the structure and properties of bioactive glasses. J Non-Cryst Solids.

[78] Hill RG, Brauer DS (2011). Predicting the bioactivity of glasses using the network connectivity or split network models. J Non-Cryst Solids.

[79] Pedone A, Malavasi G, Menziani MC, Segre U, Cormack AN (2008). Role of magnesium in soda-lime glasses: insight into structural, transport, and mechanical properties through computer simulations. J Phys Chem C.

[80] Macon ALB, Kim TB, Valliant EM, Goetschius K, Brow RK, Day DE (2015). A unified in vitro evaluation for apatite-forming ability of bioactive glasses and their variants. J Mater Sci - Mater Med.

[81] Harris KL, Bainbridge NJ, Jordan NR, Sharpe JR (2009). The effect of topical analgesics on ex vivo skin growth and human keratinocyte and fibroblast behavior. Wound Repair Regen.

[82] Oudadesse H, Dietrich E, Gal YL, Pellen P, Bureau B, Mostafa AA, et al. Apatite forming ability and cytocompatibility of pure and Zn-doped bioactive glasses. Biomed Mater. 2011;6(3). 10.1088/748-6041/6/3/035006. 10.1088/1748-6041/6/3/035006 21505231

[83] Sanders DM, Person WB, Hench LL (1974). Quantitative-analysis of glass structure with use of infrared reflection spectra. Appl Spectrosc.

[84] Jones JR, Sepulveda P, Hench LL (2001). Dose-dependent behavior of bioactive glass dissolution. J Biomed Mater Res.

[85] Vengellur A, LaPres JJ (2004). The role of hypoxia inducible factor 1 alpha in cobalt chloride induced cell death in mouse embryonic fibroblasts. Toxicol Sci.

[86] Lu X, Leng Y (2005). Theoretical analysis of calcium phosphate precipitation in simulated body fluid. Biomaterials..

[87] Fredholm YC, Karpukhina N, Brauer DS, Jones JR, Law RV, Hill RG (2012). Influence of strontium for calcium substitution in bioactive glasses on degradation, ion release and apatite formation. J Roy Soc Int.

[88] Dietrich E, Oudadesse H, Lucas-Girot A, Le Gal Y, Jeanne S, Cathelineau G (2008). Effects of mg and Zn on the surface of doped melt-derived glass for biomaterials applications. Appl Surf Sci.

[89] Ahmad NE, Fearn S, Jones JR, Lee WE, Angeli F, Delaye JM, Schuller S, Pinet O, Rebiscoul D, Gin S (2014). Preliminary surface study of short term dissolution of UK high level waste glass. 2nd International Summer School on Nuclear Glass Wasteform: Structure, Properties and Long-Term Behavior. Procedia Materials Science. 7.

[90] Kramer E, Itzkowitz E, Wei M (2014). Synthesis and characterization of cobalt-substituted hydroxyapatite powders. Ceram Int.

[91] Cailleteau C, Angeli F, Devreux F, Gin S, Jestin J, Jollivet P (2008). Insight into silicate-glass corrosion mechanisms. Nature Mater.

[92] Clark AE, Pantano CG, Hench LL (1976). Auger spectroscopic analysis of bioglass corrosion films. J Am Ceram Soc.

[93] Majerus O, Gerardin T, Manolescu G, Barboux P, Caurant D (2014). Effect of aqueous Mg2+ and Ca2+ cations on the dissolution kinetics and alteration layer of sodium borosilicate glasses at neutral pH buffered with Tris/HCI. Phys Chem Glasses - Eur J Glass Sci Technol Part B.

[94] Gerhardt L-C, Widdows KL, Erol MM, Burch CW, Sanz-Herrera JA, Ochoa I (2011). The pro-angiogenic properties of multi-functional bioactive glass composite scaffolds. Biomaterials..

[95] Detsch R, Stoor P, Grunewald A, Roether JA, Lindfors NC, Boccaccini AR (2014). Increase in VEGF secretion from human fibroblast cells by bioactive glass S53P4 to stimulate angiogenesis in bone. J Biomed Mater Res Part A.

[96] Afzali H, Kashi AHJ, Momen-Heravi M, Razzaghi R, Amirani E, Bahmani F (2019). The effects of magnesium and vitamin E co-supplementation on wound healing and metabolic status in patients with diabetic foot ulcer: a randomized, double-blind, placebo-controlled trial. Wound Repair Regen.

[97] Bernardini D, Nasulewicz A, Mazur A, Maier JAM (2005). Magnesium and microvascular endothelial cells: a role in inflammation and angiogenesis. Frontiers in Bioscience-Landmark.

[98] Wang M, Yu YM, Dai K, Ma ZY, Liu Y, Wang J (2016). Improved osteogenesis and angiogenesis of magnesium-doped calcium phosphate cement via macrophage immunomodulation. Biomater Sci.

[99] Xu L, Willumeit-Romer R, Luthringer-Feyerabend BJC (2019). Effect of magnesium-degradation products and hypoxia on the angiogenesis of human umbilical vein endothelial cells. Acta Biomater.

