# Supplementary figures and images for: Bioactive glasses and electrospun composites that release cobalt to stimulate the HIF pathway for wound healing applications
Source: Biomater Res. 2021 Jan 15;25:1. doi: 10.1186/s40824-020-00202-6 (PMC7811269; doi:10.1186/s40824-020-00202-6)

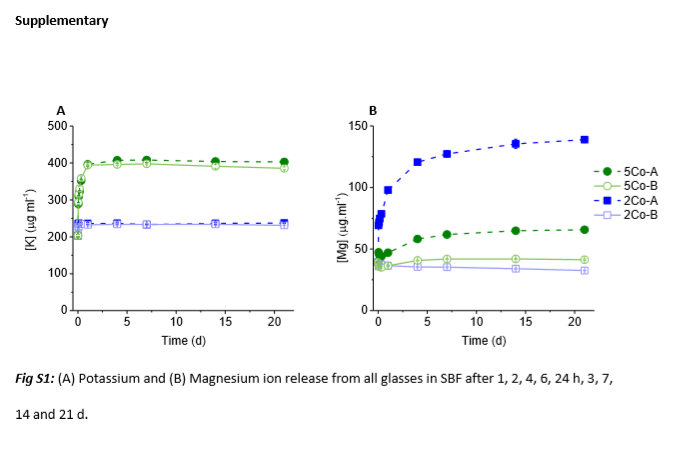


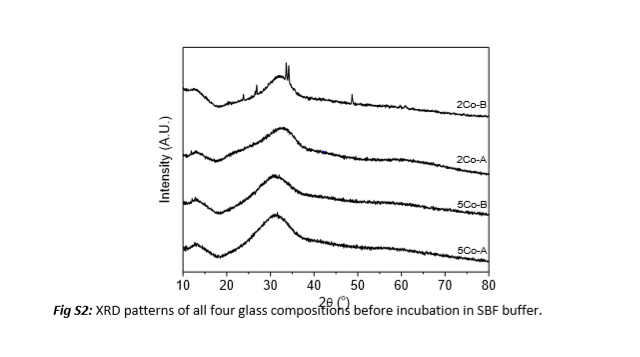


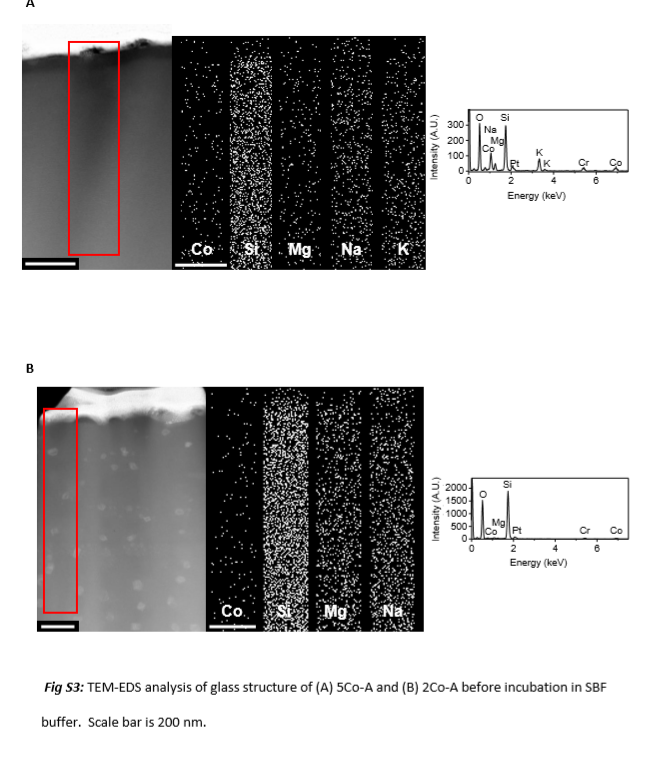


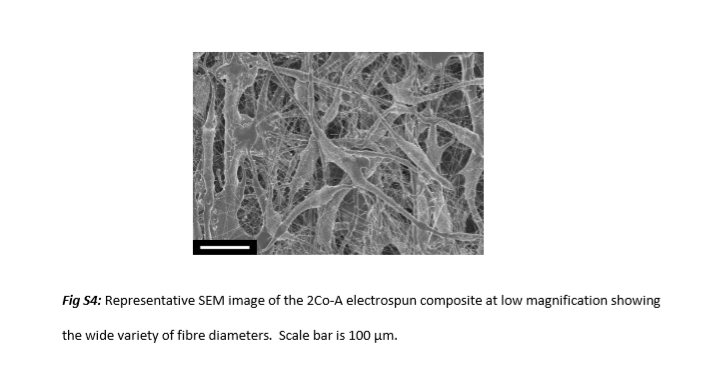

Supplement: Supplementary file 1 — Additional file 1: Figure S1. (A) Potassium and (B) Magnesium ion release from all glasses in SBF after 1, 2, 4, 6, 24 h, 3, 7, 14 and 21 d. Figure S2. XRD patterns of all four glass compositions before incubation in SBF buffer. Figure S3. TEM-EDS analysis of glass structure of (A) 5Co-A and (B) 2Co-A before incubation in SBF buffer. Scale bar is 200 nm. Figure S4. Representative SEM image of the 2Co-A electrospun composite at low magnification showing the wide variety of fibre diameters. Scale bar is 100 μm. [file 40824_2020_202_MOESM1_ESM.docx]
